# Notes and News

**Published:** 1898-02-19

**Authors:** 


### Notes and News.

(Collected and Communicated.)

### HOSPITAL MEETINGS.

Royal Free Hospital.—Annual Court, Wednesday, February 23rd, at 4.30 p.m., at the Hospital.

Royal Hospital for Women and Children.—The Annual Meeting will be held at the Mansion House, on Monday, 28th inst., at 3 p.m., the Lord Mayor presiding.

City of London Hospital for Diseases of the Chest.—Annual Court of Governors, at Gresham College, at 4 p.m., February 28th.

National Orthopædic Hospital.—Annual Meeting, Monday, February 28th, at 4 p.m., at the Hospital.

King's College Hospital.—Annual Court, Thursday, February 24th, at 4 p.m., in the Board-room of the Hospital.

Royal Sea-Bathing Infirmary, Margate.—A Special Court of Governors will be held at 30, Charing Cross, on Friday, February 18th, at 3 o'clock.

East London Hospital for Children.—Annual Court, Wednesday, February 23rd, at 2.30 p.m., at the Hospital.

West Ham Hospital.—Annual Meeting, Wednesday, March 9th, at 8 p.m., in the Dispensary Hall.

St. John's Hospital for Diseases of the Skin.—Annual General Meeting, Friday, February 25th, at the Hotel Victoria, 4.30 p.m.

Out of the legacy of £2,200 left by the late Mr. Croft to the Grimsby Hospital, the sum of £1,100 has to be deducted for estate duties.

The Bishop of London will preside at the dinner at the Hotel Metropole, on April 28th, on behalf of the Great Ormond Street Children's Hospital, when a special appeal will be made towards a fund of £30,000 for the purchase of the adjoining grounds and Convent of St. John and St. Elizabeth. It is hoped the convent building, with its 40 bedrooms and beautiful oak-lined refectory, will form the future Nurses' Home of the Ormond Street Hospital.

On the 10th inst. the annual meeting of the supporters of the Asylum for the Deaf and Dumb took place at the City Terminus Hotel. Twenty children, 12 boys and 8 girls, were elected, and the excess of expenditure over income was shown to reach £3,590. This institution has about 350 children in its care at the institutions in the Old Kent Road and at Margate. Since its foundation in 1792 more than 5,000 deaf mutes have been assisted.

The annual report of the Ventnor Hospital for Consumption is very satisfactory. The new block of buildings, of which the foundation-stone was laid last year by Princess Henry of Battenberg, will soon be completed, bringing the number of beds at the hospital up to 155. Mr. P. B. Burgoyne has been elected treasurer.

The Executive Committee of the Bengal branch of the Countess of Dufferin's Fund are asking for subscriptions for a new Zenana Hospital in Calcutta. The former hospital—the Victoria—was sold, since the rapid growth of the city made the site too crowded and noisy. The foundation-stone of the new hospital was laid on January 14th by the Countess of Elgin.

The Keighley Hospital is to be considerably enlarged, plans for the improvement having been passed. An entirely new administrative block is to be added, which will furnish accommodation for private and district nurses. A two storey-building adjoining will contain ward accommodation to bring the capacity of the hospital up to 100 beds.

There is a deficit on the year in the funds of the Radcliffe Infirmary, Oxford, amounting to £600. At the last Court it was decided to make extensive alterations in the old block of buildings, and to provide a new operating theatre, these improvements to cost £4,370. Lord Jersey, presiding, spoke of the necessity of these alterations, stating that, if matters were allowed to go on as at present, they "would almost amount to a scandal."

At a full meeting of the executive committee of the Bristol centre of the St. John Ambulance Association, the following resolution was passed: "That the executive committee think that the proposed employment of lecturers other than medical men (who have always hitherto officiated in that capacity) to conduct classes on hygiene in connection with the home hygiene course is much to be deprecated, and cannot be carried out without loss of prestige to the St. John Ambulance Association."

The *Rangoon Times* protests in the public interests against the patients of the Rangoon Leper Asylum being granted leave of absence in order to engage in reaping food crops. The object of the asylum is to segregate lepers, and the *Rangoon Times* says, "It is loathsome to think of lepers being permitted to handle the food of the people, for though scientific opinion differs as to the contagious nature of leprosy, it is repulsive to sight and feelings, and the public subscribed to this leper asylum partly to thin the district of Burma from the presence of wandering mendicant lepers."

Dr. Erskine recently gave an address before Anderson's College Medico-Chirurgical Society in Glasgow on "The Hospitals of Russia." He said there were no hospitals worthy of the name in the Empire till Peter the Great built one in Moscow in 1707 after the exact pattern of a hospital he had seen in Greenwich. In Russia a body of men called Feldschers exists who are half doctors and half nurses. They undergo a course of training and obtain a diploma, are far more numerous than qualified practitioners, and, in Dr. Erskine's opinion, are indispensable to the peasantry in outlying districts.

